# Bio-Inspired New Hydraulic Actuator Imitating the Human Muscles for Mobile Robots

**DOI:** 10.3389/fbioe.2022.923383

**Published:** 2022-06-27

**Authors:** Xiangjuan Bai, Yong Xue, Yuze Xu, Jianzhong Shang, Zirong Luo, Junhong Yang

**Affiliations:** ^1^ College of Intelligence Science and Technology, National University of Defense Technology, Changsha, China; ^2^ Beijing Special Engineering Design Institution, Beijing, China; ^3^ DIT Group Limited (DIT), Changsha, China

**Keywords:** mobile robots, bionic robots, bionic actuators, hydraulic systems, Efficency, Muscle

## Abstract

Limited load capacity is the bottleneck for the practical application of mobile multi-joint legged robots. And improving the efficiency of the drive system is a key factor in improving the load capacity. To improve the efficiency of mobile robots, in this paper, a new kind of actuator that imitates the driving mechanism of human muscles is innovatively designed and validated through experiments. The proposed actuator consists of a single power source and multiple plunger pistons, and imitates the configuration of a human muscle, to improve the efficiency and load capacities. The design proposed here represents a new class of driving methods. The actuator selects the most appropriate combination of the effective areas of plunger pistons like the human muscles, to ensure that the maximal output force aligns with the load force. To validate that the new actuator can improve the efficiency of hydraulic systems of mobile robots, a robotic arm incorporating a prototype of the new actuator was designed. The proposed system was validated through a series of experiments. The experiments show that the bionic actuator can adjust the flow rate of the system input by adjusting the number and size of the motion units involved in the work, and with the change in load force, it changes the output force by recruiting different motion units, which indicates good controllability. The results reported herein reveal that the application of bionics to the design of robotic actuator can significantly improve the efficiency and overall performance of the robots, and this biomimetic approach can be applied to a variety of robots.

## 1 Introduction

With the development of human perception, information fusion, and bionic control technologies, the performance of the actuator structures in bionic robots is constantly improving. To increase the system power density of the biomimetic robots, and consequently, their load capacity, many researchers have attempted to substitute the hydraulic actuator (HA) systems with other actuator systems (Amundson et al., 2006), such as flexible actuators and novel material actuators. HA systems are relatively better than the artificial muscles made of soft materials in terms of load capacity (Lin et al., 2021a; Lin et al., 2021b). Therefore, the hydraulic systems used in the bionic actuator structures indicate an important research direction in the field of bionic robotics. Some examples include the typical BigDog, WildCat, and SpotMini designed by Boston Dynamics Company (Semini et al., 2011; Davis, 2013), and the Cheetah designed by MIT (Bledt et al., 2018). In relevant research, Durfee et al. discovered that under the same power source condition, when the system pressure exceeded 3.5 MPa, the HA system possessed a higher power density than the electromechanical drive system (Durfee et al., 2011). However, owing to the limitations of volume and weight, a single pump-source hydraulic system is generally adopted for most hydraulic systems of bionic robots (Roozing et al., 2016; Yang et al., 2019). Since a load of each actuator does not match the output pressure of the pump source, significant throttling loss occurs, which in turn reduces the efficiency of this type of hydraulic pressure (J and M, 2011; Szabó et al., 2019; De Keyser et al., 2021). In general, the load of a biomimetic robot actuator varies drastically with time and working state, therefore, a single cylinder cannot satisfy the dynamically changing system load. Moreover, a low-efficiency hydraulic system increases the overall weight of the entire structure and causes other problems, such as excessive energy consumption, reduced battery life, and system overheating (Busquets and Ivantysynova, 2015). In addition, the environmental concerns stemming from the energy consumption demand that hydraulic systems be more energy-efficient (H and F, 2013; Awad et al., 2020). The energy efficiency of the mobile robots is generally evaluated based on the total cost of transport (COT) (Seok et al., 2013), which is inversely proportional to the energy efficiency. The COTs of BigDog and Cheetah robots are 15 and 0.51, respectively, whereas that of the human body is only 0.2 (Seok et al., 2013; Seok et al., 2015). Hence, by deploying the principle of bionics and imitating the human energy supply system, muscle structure, and adjustment method, the muscle’s output force can certainly aid in achieving a high energy efficiency (Zoss et al., 2006; Furnemont et al., 2016; Liang et al., 2020).

With recent advances in computer technology, using digital hydraulic components and their control to improve the efficiency of hydraulic systems has now become a research hotspot (M., 2000). For instance, the direct current transformer controls the on-off valve by adjusting the pressure (Axin et al., 2013) and the flow of the hydraulic system through pulse width modulation (PWM) (M., 2000; Guglielmino et al., 2010; Bhatti and Plummer, 2011). Progressively, Kyoung Kwan Ahn et al. of Ulsan University proposed the use of accumulators to recover the energy and release the hydraulic system (Ho and Ahn, 2010; 2012). In addition, researchers have also proposed the reduction in energy consumption by controlling the supply pressure of the system (M., 2000; Baghestan et al., 2015; Du et al., 2016).

Scientific experts have demonstrated the possibility of improving the efficiency of hydraulic systems using several methods (Altare and Vacca, 2015). To increase the power density of the power source, researchers used gasoline engines, such as the two-stroke gasoline engine used by BigDog (Davis, 2013) and the hydraulic drive structure of exoskeleton equipment, as the power source to drive pumps that power the hydraulic system of the robot. Such a type of driving method to improve efficiency is generally used in robots with large loads or large sizes. Alternatively, some scholars have also proposed a chemical reaction based power source for the biomimetic robots in specific environments. Meanwhile, other scholars attempted to improve the power density using highly integrated hydraulic actuators (Kim et al., 2020). Likewise, a highly integrated HA was designed for a bionic robot (Altare and Vacca, 2015), and then, a miniature HA system comprising a pump and a finger joint hydraulic unit was designed for the palm prosthesis (Kargov et al., 2008). To improve efficiency, this type of driving method is widely used in small and medium-sized bionic robots. Furthermore, using special materials and energy recovery methods, as well as through the application of digital transformers, discrete adjustment of different working chamber combinations, and electro-hydraulic hybrid drive (Shenouda, 2006; Bai et al., 2022), the energy efficiency of the hydraulic system has been improved (Eriksson and Palmberg, 2011). This approach is commonly employed in micro-robots or micro-nano robots, due to its relatively small driving force.

Although all these approaches can improve the power density of the HA system to a certain extent, they are unable to improve the efficiency of mobile multi-joint legged robot actuators. Since the load characteristics of such a robot change randomly with time, it requires a small size, and the high-power density and efficiency of the driving characteristics are desirable. In this regard, the development of new and efficient driving methods can make the bionic multi-joint legged robots more energy-efficient, and thus, designing advanced bionic actuators for the multi-joint legged robots is highly critical.

For the bionic robot, its design inspiration comes from the shape structure, and motion law of humans and animals, as well as its energy supply. The energy consumed by a human or an animal to complete the same action is much smaller than a bionic robot. This low energy consumption is not only related to the shape structure and movement laws of humans or animals, but also to the way of energy distribution and supply, the structure of muscle actuators, and the way they adjust their output force. Therefore, imitating the energy supply, muscle structure, and regulation of output force of humans or animals is one of the effective ways to break through low efficiency, which restricts robots to practical use. Accordingly, in this study, a new type of bionic hydraulic actuator (BHA), with a variable effective area, is designed to improve the efficiency of the hydraulic system of bionic robots (especially for mobile multi-joint robots, such as quadruped robots, biped robots, etc.). The BHA takes the mechanical arm as the experimental application object to carry out experimental verification. The BHA can also be widely used in other types of bionic multi-joint mobile robots. The design of the new BHA relied on the driving principle of human muscles. Furthermore, by imitating the structure of human muscles, the BHA was designed using a pump and multiple piston chambers. This BHA can change the output force by varying the flow rate of the hydraulic pump, to match different load forces. A more integrated BHA can avoid complex tubing and connections, while also reducing size and mass. The newly designed BHA is highly efficient and can be deployed in mobile bionic robots with time-variant load force.

The remainder of this paper is organized as follows. The principle of the new actuator based on imitation of the human muscle is presented in Section 2. Hydraulic systems with traditional and new actuator are presented in Section 3. The efficiency of both actuators is compared based on a robotic arm, and the findings are detailed in Section 4. In Section 5, the performance of the newly designed actuator is validated using an experimental system. Finally, the conclusions and future work are presented in Section 6.

## 2 Principle of the New Actuator Based on Imitation of the Human Muscle

**FIGURE 1:**
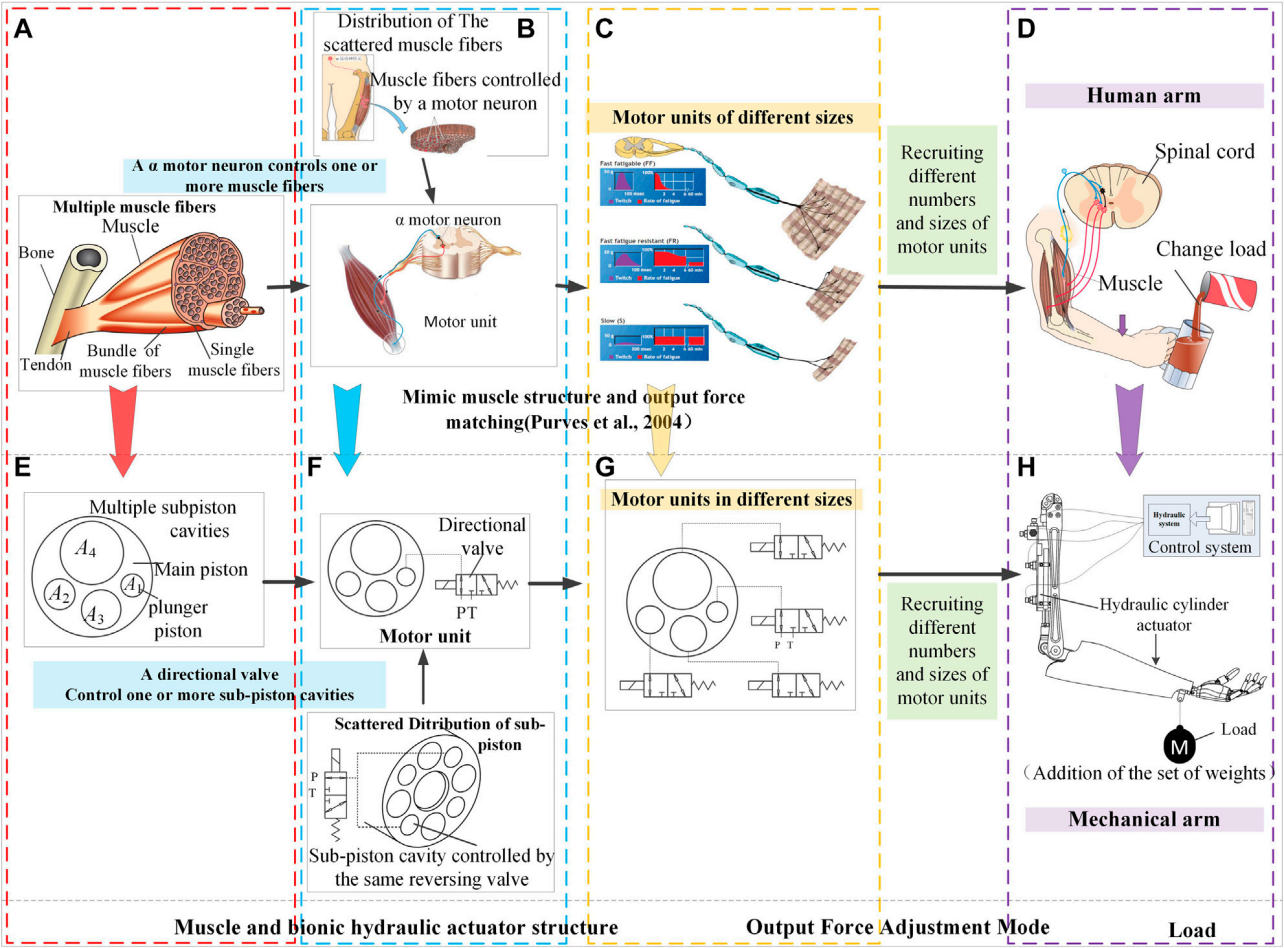
Biomimetic design mechanism of a new bionic hydraulic actuator by imitating human muscles; **(A)** to **(E)**: Mimic the structural characteristics of human muscle multi-motor units; **(B)** to **(F)**: Mimic the control of human muscles and dispersion and distribution of a muscle motor unit as closely as possible on the cross-section; **(C)** to **(G)**: Mimic the scale difference of human muscle motor units and the recruitment patterns of muscle motor units; **(D)** to **(H)**. Comparison of human arm and robotic arm.

As shown in Figure 1D, all the motions of human skeletal joints are executed by the expansion and contraction of the muscles on skeletal muscles. The human brain transmits action instructions to the spinal cord motor neuron that controls muscle contraction and stretching. For muscles, the bottom layer is the output force of muscle fibers controlled by motor nerves.

When the human performs different load tasks, the muscle driving force of each skeletal joint is required to be matched in real-time with the load. The excellent structural characteristics of skeletal muscle and its control methods can be summarized as follows:

1. The structural characteristics of human muscle:

A skeletal muscle contains multiple motor units, as shown in Figure 1A. Skeletal muscle is composed of many muscle fibers of different sizes, which form different muscle bundles, each of them is innervated by α motor neuron. An α motor neuron and its corresponding multiple muscle fibers together constitute the smallest motor unit. The existence of multiple motor units in a muscle is the main structural feature to ensure the output force of the muscle. In addition, it can avoid affecting the exercise capacity of the entire muscle due to the failure of one or several motor units (Purves et al., 2004).

2. The dispersion and distribution of a muscle motor unit:

The output force of a motor unit is relatively dispersed across the muscle. A muscle bundle is distributed in a large area. This distribution feature can make the output force distribution of the motor unit relatively uniform, which is beneficial to the superposition of the output force of different motor units.

3. The control of human muscles:

Muscle expansion and contraction and changes in output force are directly controlled by α motor neurons in the spinal cord. Brainstem and cerebellum control of muscles is also achieved by sending motor commands to α motor neurons (Purves et al., 2004). The relationship between α motor neurons and muscles is shown in Figure 1B. The excitation or inhibition of α motor neurons can stimulate the contraction and stretching of the muscles it innervates, thereby driving the body’s skeletal movements.

4. The scale difference of human muscle motor units:

There are also differences in scales between motor units and their control neurons, and the corresponding output forces of motor units at different scales are also different, as shown in Figure 1C.

5. The recruitment patterns of muscle motor units:

Muscle bundles of different sizes form differently sized motor units to correspond to different sizes of output force, as shown in Figure 1C. Fundamentally, small motor units drive a small output force while large motor units drive a large output force, thereby driving human bones to perform different types of motion (Purves et al., 2004). Moreover, there are different types of nerve fibers in the muscle bundles (Xue et al., 2015). The motor units of most skeletal muscles are divided into the categories of slow speed (S), fast fatigue (FF), and fast fatigue resistance (FR). Slow motor muscle fibers produce less force, whereas the FF muscle fibers are composed of large muscle fibers that yield greater strength (running or jumping) and have a suitable instantaneous explosive power. Lastly, the fast FR muscle bundle is not significantly large and possesses a medium speed. Through real-time adjustment of the number of muscle bundles involved in supplying the output force, different human actions can be realized. The number of muscle bundle motor units is adjusted in a fixed order, according to the required output force. That is, as the required muscle output force changes from a small value to a large value, the corresponding motor unit changes from S unit, FR unit to FF unit.

**FIGURE 2:**
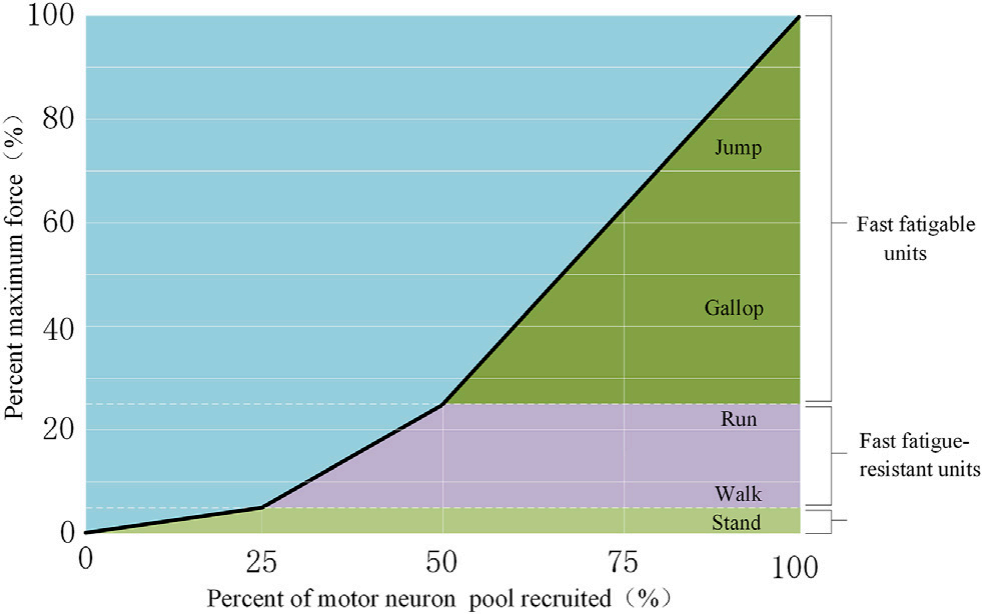
Recruitment of motor neurons for different movements (Purves et al., 2004).

For example, a cat’s medial gastrocnemius muscle operates according to the above principles (Purves et al., 2004), as shown in Figure 2. When the cat is quiet, only a small amount of force (approximately 5%) is required from the muscle bundle. At this time, the S unit provides the output force, which accounts for approximately 25% of the motor unit in the muscle. When the cat walks slowly (prior to running), the output force required from the muscles is approximately 25% of the total muscle strength, and the FR unit is sufficient for such a case. Similarly, the FF unit is used when the cat performs a significant amount of activity, such as running or jumping. Throughout the process, the muscle bundle motor unit provides the muscle power, according to the required output force.

The actuators were required to output different driving forces for different loads while performing different load tasks. As shown in Figure 1D, the muscle must output larger forces to drive the arm, when more liquid is added to the glass. In skeletal muscles, there are multiple motor units composed of motor muscle bundles, each of which includes the multiple muscle fibers that can perform an exercise and the corresponding control neurons (Purves et al., 2004). These multiple motor units realize the action of the muscle force and drive the bone movement in the human body.

Therefore, imitating the bionic mechanism (configuration and control mechanism) of humans or animals is the key to the design of the BHA. The design mechanism is shown in Figure 1. Figure 1H demonstrates a robotic arm driven by a linear HA similar to a muscle of the human body. The control system of the robotic arm was composed of an electronic control system and a hydraulic system. According to the action required by a robot, instruction signals were converted into control signals of the hydraulic system, to control the HA for driving the robotic arm.

When the robotic arm performs the tasks with different loads, as shown in Figure 1H, the HA must output varying forces according to the load. For a constant effective area hydraulic actuator (CHA), that is, a traditional hydraulic cylinder actuator, this can only be achieved by changing the pressure of the oil flowing into the chamber of the actuator. The driving force of a traditional cylinder actuator is expressed as:

$$F=P_{l}A_{l}-P_{r}A_{r}$$ (1)

where $P_{l}$ and $P_{r}$ are the oil pressures in the right and left chambers of the cylinder, and $A_{l}$ $A_{r}$ are the effective areas of the right and left chambers, respectively.

However, the depression of oil pressure can trigger unnecessary energy usage, specifically when the robotic arm performs a task with numerous loads. Based on Eq. 1, if the effective area of the actuator is variable, the force can also be changed while maintaining the constant pressure of the oil.

**FIGURE 3:**
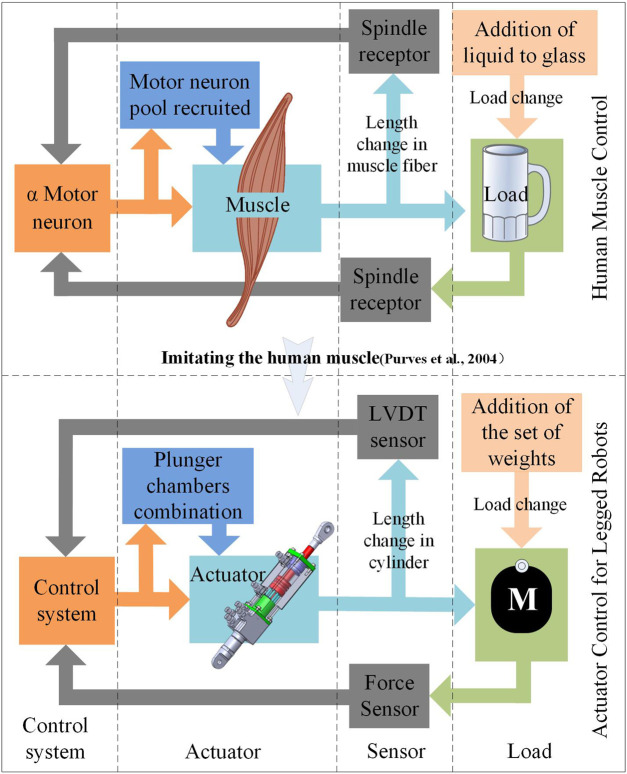
Control mode of the muscle and new actuator.

In this way, the size principle of the human muscle is incorporated into the design of the new actuator. Additionally, to imitate the configuration of human muscle, the proposed actuator is designed with multiple different plunger pistons, as shown in Figure 1E. Moreover, Figure 3A presents the configuration of the muscles. The muscle comprises many bundles of muscle fibers, each of which comprises a single muscle fiber. By maintaining a constant level of oil pressure, the actuator can also output different forces by recruiting the effective areas of plunger pistons in a fixed order based on their dimensions.

Muscles can match the output force and load by orderly recruitment of motor units of varying scales. Hence, by imitating the structural characteristics, and using the muscle recruitment method of motor units, the problem of the inability of traditional bionic robot HA to adjust their maximum output force according to the change of load force, which yields low energy efficiency, can be solved. A BHA design mechanism for multi-motor unit and multiple different plunger pistons is proposed, as shown in Figure 1. The design mechanism is described as follows:

1) Mimic the structural characteristics of human muscle multi-motor units: The BHA is designed as a cylinder with multiple plunger pistons, as shown in Figure 1E. Each piston chamber is connected to a two-position three-way switch valve that determined whether the connected piston chamber is connected to a high-pressure oil circuit or a low-pressure oil circuit, as shown in Figure 1F. The switch valve corresponds to the α motor neuron, while the piston cavity corresponds to the muscle fiber. Together, the switch valve and the piston cavity constitute a motor unit.
2) Mimic the scale difference of human muscle motor units: For muscles, the size of a motor unit is directly proportional to the amount of output force, as shown in Figure 1G. In contrast, for the BHA, the piston chamber cut-off area controlled by the small-diameter switch valve is small, resulting in a small motor unit with a small output force, when high-pressure oil is used for the connection. On the other hand, the large piston cavity cut-off area controlled by the large-diameter switch valve is large, thereby resulting in a large motor unit with a large output force, when high-pressure oil is used for the connection.
3) Mimic the dispersion and distribution of a muscle motor unit as closely as possible on the cross-section: The position of each piston cavity in the cross-section of the entire multi-cavity BHA is dispersed symmetrically along the circumference as widely as possible, to superimpose the output force of each piston cavity on the output shaft of the entire multi-cavity BHA, as shown in Figure 1G.
4) Mimic the recruitment patterns of muscle motor units: By controlling the switch valve, different piston chambers of the actuator are recruited to participate in the activities of a motor unit, thereby obtaining different output forces and ensuring that the output force matches the load.

The upper part of Figures 1A–D illustrates the control process of the muscle when the human arms perform variable-load tasks. The spindle receptor can sense the length change in the muscle fiber and provide direct feedback to the motor neuron (Purves et al., 2004), constituting a position closed loop. When the liquid is poured into the glass, the spindle receptor can sense the change in muscle force and provide feedback to the motor neurons, to recruit more motor units, as long as the muscle force is sufficient for the load. Alternatively, the control process of the proposed actuator, relying on a combination of the characteristics of the hydraulic system and the imitation of the control mode of the muscle, is shown in the lower part of Figures 1E–H. Likewise, the control system comprised an electronic control system and a hydraulic system, as depicted in Figure 3. When the system controls the actuator to drive the robotic arm, the length of the cylinder can be sensed using a linear variable differential transformer (LVDT) displacement sensor that sends feedback to the control system.

Concurrently, when the load was changed, the force sensor could provide feedback signals to the control system, in order to determine the most appropriate combination of effective areas of the plunger pistons.

## 3 Hydraulic System With the Traditional and Proposed Actuator

In this section, the hydraulic systems based on the traditional and proposed actuators are presented, and the energy consumption of both systems is expressed using mathematical equations.

### 3.1 Traditional Actuator

**FIGURE 4:**
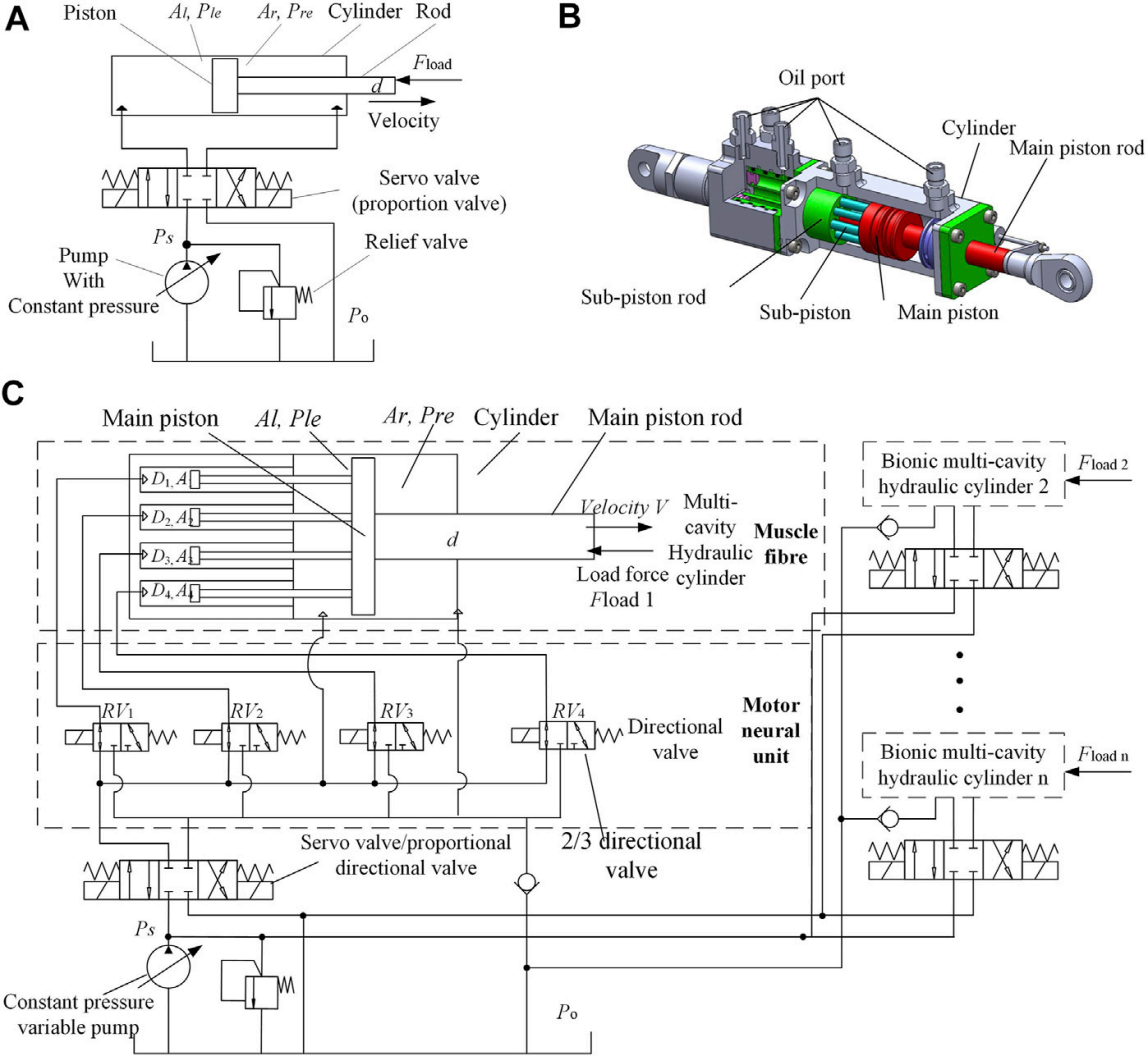
Design of a New Biomimetic Hydraulic Actuator **(A)**. Hydraulic actuator system with typical cylinder; **(B)**. Multi-chamber hydraulic actuator structure; **(C)**. Hydraulic system structure based on the bionic hydraulic actuator.

Currently, most legged robots are actuated using a typical hydraulic system, as shown in Figure 4A, where a joint is actuated by one or more typical hydraulic cylinders. Such hydraulic systems consist of four-way electrohydraulic proportional valves and actuators and are supplied with a pump in parallel with a relief valve.

Assuming the oil is rigid, the rate of oil flowing into the rod-less chamber $A_{l}$ and rod chamber $A_{r}$ is expressed as:

$$Q_{l}=v_{l}S_{l}.$$ (2)

$$Q_{r}=v_{r}S_{r}.$$ (3)

where $Q_{l}$ and $Q_{r}$ denote the rate at which the oil flowed into the rod-less and rod chambers, with areas $S_{l}$ and $S_{r}$ , respectively (Xue et al., 2015). Meanwhile, $v_{l}$ and $v_{r}$ are the velocities of the piston rod extension and retraction, respectively.

All the input power was supplied using the high-pressure source $P_{s}$ . In addition, energy consumption is defined as a periodic process consisting of two sub-processes, namely, rod extension and retraction. The rod extension and retraction times were defined as $T_{e}$ and $T_{r}$ , respectively. Consequently, the energy consumption can be expressed as follows:

$$E_{1}=\int_{0}^{T_{e}}v_{l}S_{l}P_{s}dt+\int_{0}^{T_{r}}v_{r}S_{r}P_{s}dt.$$ (4)

### 3.2 New Actuator With a Variable Effective Area

Using the above-mentioned design mechanism, different types of multi-motor units and multi-chamber BHA can be designed, according to the specific requirements of the project.

Based on the proposed bionic hydraulic cylinder structure, three distinct hydraulic cylinder-actuator prototypes can be conceived. They can be of asymmetrical structure type, symmetrical structure type, and nested structure type, respectively. This work considers the symmetrical structure of the bionic multi-chamber hydraulic cylinder actuator, in designing and subsequent experimentation. That is to say, the sub-pistons of the symmetrical actuator structure are distributed symmetrically.

Figure 4B shows a sample multi-cavity hydraulic cylinder and its designed motor unit recruitment mode based on this mechanism. The difference between this structure and the CHA structure is that the main piston rod of the multi-cavity hydraulic cylinder here is connected to multiple sub-piston rods, where a sub-piston is connected to the sub-piston rod, and the sub-piston is arranged in the sub-piston cavity to follow the reciprocating movement of the main piston. The sub-piston and main piston divide the inner cavity of the BHA into multiple chambers, each with an inlet and outlet, as demonstrated in Figure 4B. Obviously, the larger the size of the hydraulic cylinder, the more the sub-pistons that can be accommodated, the more the adjustable effective action area, and the better the load matching. However, the system with a larger number of sub-pistons (where the volume of each sub-piston is smaller) is more difficult to process and manufacture; hence, it is necessary to choose an appropriate number of sub-pistons. In this design, the BHA is composed of a cylinder, four sub-pistons, sub-piston rods, and one main piston and one main piston rod. The main piston and the four sub-pistons are firmly connected using the four sub-piston rods. The BHA contains six cavities, among which there are four sub-piston cavities ( $A_{1}$ $A_{2}$ , $A_{3}$ , and $A_{4}$ ), one cavity $A_{l}$ on the left, and one-rod cavity $A_{r}$ on the right. These six cavities with different effective areas are analogous to six fiber bundles of different sizes in the human muscles. In addition, the switch valve connected to the sub-piston cavity is equivalent to the α-motor neural unit used in muscle movement. It follows the scale principle of the α-motor neural unit and muscle fibers innervated by it. Moreover, the cavities with small and large effective areas correspond to small and large switch valves, respectively. Essentially, the switch valve and the cavity controlled by it constitute a motor unit.

Furthermore, four switch valves control four cavities to form four different scale motor units in this considered design example. Through the combination of different switch valves, various motor unit recruitment methods can be obtained, thereby achieving various output forces. By selecting different motor unit recruitment methods, the output force and the load can be matched, effectively. Notably, the motor units in the human muscles significantly outnumber the motor units used in this design example. According to the principle of permutation and combination, muscles have numerous different motor unit recruitment methods, and their output force can be regarded as continuously adjustable. This design example, however, has only four motor units, and the output force can achieve 16 variations. Nevertheless, it significantly reduces the throttling loss caused by the difference between the output and the load forces. Although the effective area of BHA can be changed by recruiting different motor units, this change can only be discrete owing to the limited number of sub-pistons. To achieve a precise matching, a servo or a proportional and directional valve is used at the high-pressure outlet of the pump, as shown in Figure 4C. This can fine-tune the outlet pressure of the pump, and simultaneously enable the BHA to achieve high-precision position and force tracking. The control of four switching valves is defined as $x=\left[\begin{array}{cccc} x_{1} & x_{2} & x_{3} & x_{4} \end{array}\right]$ , where $x_{k}=1\left(k=1∼4\right)$ indicates that the chamber $A_{k}$ is connected to the left chamber $A_{l}$ ; while $x_{k}=0$ indicates that the chamber $A_{k}$ is connected to the right chamber $A_{r}$ . $S_{k}$ is the area of the piston chamber, $A_{k}$ , and $S_{l}$ is the area of the left chamber, $A_{l}$ , which is the area of the main piston excluding some areas ( $S_{1}$ , $S_{2}$ , $S_{3}$ , and $S_{4}$ ). Likewise, $S_{r}$ is the area of the right chamber, $A_{r}$ , which is the area of the main piston excluding the area of the main rod (Xue et al., 2015). That is to say, the relationship between the left effective area ( $S_{le}$ ), right effective area $S_{re}$ , and switching valve control x can be expressed as follows (Xue et al., 2015):

$$S_{le}=S_{l}+\sum_{k=1}^{4}S_{k}x_{k}.$$ (5)

$$S_{re}=S_{r}+\sum_{k=1}^{4}S_{k}\left(x_{k}-1\right).$$ (6)

The left and right pressures are $p_{le}$ and $p_{re}$ , respectively. Meanwhile, the friction force is f , and the output force of the BHA can be obtained as follows:

$$F_{op}=p_{le}S_{le}-p_{re}S_{re}-f.$$ (7)

Progressively, (5), (6), and (7) can be combined as follows:

$$F_{op}=p_{le}S_{l}-p_{re}\left(S_{r}-\sum_{k=1}^{4}S_{k}\right)+\left(p_{le}-p_{re}\right)\sum_{k=1}^{4}S_{k}x_{k}-f.$$ (8)

If the pressure drop loss at the two-position three-way reversing valve port is considered and the dynamic characteristics of the valve are neglected, then, (9) can be obtained using the orifice flow equation as follows:

$$Q_{k}=C_{sw}A_{sw}\sqrt{\frac{2}{\rho }\left|\Delta P_{k}\right|}.$$ (9)

$$Q_{k}=S_{k}v.$$ (10)

Where $Q_{k}$ corresponds to the flow into or out of the cavity $A_{k}$ , $C_{sw}$ represents the flow coefficient, $A_{sw}$ is the opening area of the directional valve, and $\Delta P_{k}$ refers to the pressure drop at the *k*th directional valve port.

Using (9) and 10, the pressure drop at the directional valve port can be obtained as:

$$\Delta P_{k}=\mathrm{sgn}\left(v\right)\cdot 2\rho {\left(\frac{S_{k}v}{C_{sw}A_{sw}}\right)}^{2}.$$ (11)

Next, by adding the pressure drop loss of the two-position three-way directional valve port, the output force $F_{op}$ of the HA can be obtained as:

$$F_{op}=p_{le}S_{l}-p_{re}\left(S_{r}-\sum_{k=1}^{4}S_{k}\right)+\left(p_{le}-p_{re}\right)\sum_{k=1}^{4}S_{k}x_{k}-\sum_{k=1}^{4}\Delta P_{k}x_{k}-f.$$ (12)

Since the rod extension and retraction times are $T_{e}$ and $T_{r}$ , respectively, the energy consumption can be expressed as:

$$E_{2}=\int_{0}^{T_{e}}v_{l}S_{le}P_{s}dt+\int_{0}^{T_{r}}v_{r}S_{re}P_{s}dt.$$ (13)

### 3.3 New Actuator Load Matching Control

The hardware composition of the (BHA) control system primarily includes a hydraulic control part and an electronic control part. The hydraulic control part here mainly contains the pump station, flow meter, pressure gauge, servo valve, reversing valve group, and multi-chamber hydraulic cylinder actuator. Meanwhile, the electronic control part consists of an industrial computer, data acquisition card, reversing valve controller, servo valve driver board, force sensor, and its signal amplifier, displacement sensor and its signal amplifier, etc. During the signal acquisition in the experimental system, the current state is first passed to the main control computer through a sensor and a data acquisition card. The main control computer provides the best force matching control method by analyzing the control amount and position control parameters of the reversing valve. Then, the force matching control signal is input to the directional valve controller via a data acquisition card, to control the two-position three-way directional valve, thereby realizing the recruitment of different motion units and changing the maximum output force of the actuator. At the same time, the position control signal is also input to the servo drive board through a data acquisition card, where the opening of the servo valve is adjusted with the servo valve drive board, to achieve the position tracking. In this way, the overall control of the BHA is achieved through a combination of force matching and position tracking.

In this process, the most critical part is the load matching control of the actuator. The key to efficient load matching in bionic multi-chamber hydraulic actuators is how to select the best combination of piston chambers based on the changes in load force. The following points should be considered while designing a load matching controller:

1) Determine the effective cross-sectional area of the BHA that is closest to the load variation range. In general, to minimize throttling losses, the cross-sectional area of the BHA is selected according to the upper limit of the adjustment level in which the load force is located. However, based on such selection criteria, there can be a situation where even if the proportional servo valve port is fully opened; it still cannot meet the requirements of tracking error. This is because the selected effective cross-sectional area is not enough, and the load driving capacity of BHA is insufficient. In order to meet the position tracking error requirements, a larger cross-sectional area needs to be selected, even if it reduces the efficiency.
2) Predict the trend of position tracking error and adjust the effective cross-sectional area. If the absolute value of the error becomes smaller than the set allowable value at the next sampling instant, then keep the effective cross-sectional area of the hydraulic cylinder unchanged. On the contrary, if the absolute value of the error is greater than the allowable value at the next sampling instant, then it is necessary to further identify whether the error is positive or negative. The positive error indicates that the driving capacity of the hydraulic cylinder is insufficient, and the effective cross-sectional area of the hydraulic cylinder needs to be increased. Alternatively, the negative error corresponds to a too large cross-sectional area selection that needs to be reduced.
3) When adjusting according to the above-mentioned scheme, two issues should be considered. First, at the next moment, the PID controller itself gives a large error, and according to the above adjustment scheme, it is necessary to adjust the effective cross-sectional area. However, the error will gradually become smaller under the adjustment of the PID. The second issue is how to determine the amount of adjustment when the cross-sectional area needs to be adjusted.

By imitating the recruitment mode of human muscles, combined with the design principle of the multi-chamber BHA, the adjustment level of the output force of the BHA and the control signal of the reversing valve are established, as shown in Table 1. The BHA has four sub-pistons. According to the recruitment mode adopted by the muscles and the effective area combination of the multi-chamber BHA, whether the hydraulic cylinder is retracted or extended corresponds to 16-grade changes, that is, the control signal $x=\left[\begin{array}{cccc} x_{1} & x_{2} & x_{3} & x_{4} \end{array}\right]$ of the reversing valve has 16 combinations. Different combination methods make the BHA have different activity levels. Under the condition of constant hydraulic oil pressure, the output force of the actuator can be changed. The adjustment level of the BHA and the control signal of the reversing valve are ordered correspondingly, as shown in Table 1. When adjustment level K=1 , the effective cross-sectional area is at the maximum value, and the states of reversing valve control signal $x_{k}$ are all 1, that is, $\left[x_{1}x_{2}x_{3}x_{4}\right]=\left[1111\right]$ . The effective cross-sectional area gradually decreases with the increase K . Similarly, when K=16 , the effective cross-sectional area is the smallest, and the control signals of the reversing valve $x_{k}$ are all 0, that is $\left[x_{1}x_{2}x_{3}x_{4}\right]=\left[0000\right]$ .

**TABLE 1:** Adjustment level and control signal of directional valve.

Level K	Reversing Valve Control Signal $\left[x_{1}x_{2}x_{3}x_{4}\right]$	Level K	Reversing Valve Control Signal $\left[x_{1}x_{2}x_{3}x_{4}\right]$
1	[1111]	9	[1110]
2	[0111]	10	[0110]
3	[1011]	11	[1010]
4	[0011]	12	[0010]
5	[1101]	13	[1100]
6	[0101]	14	[0100]
7	[1001]	15	[1000]
8	[0001]	16	[0000]

**FIGURE 5:**
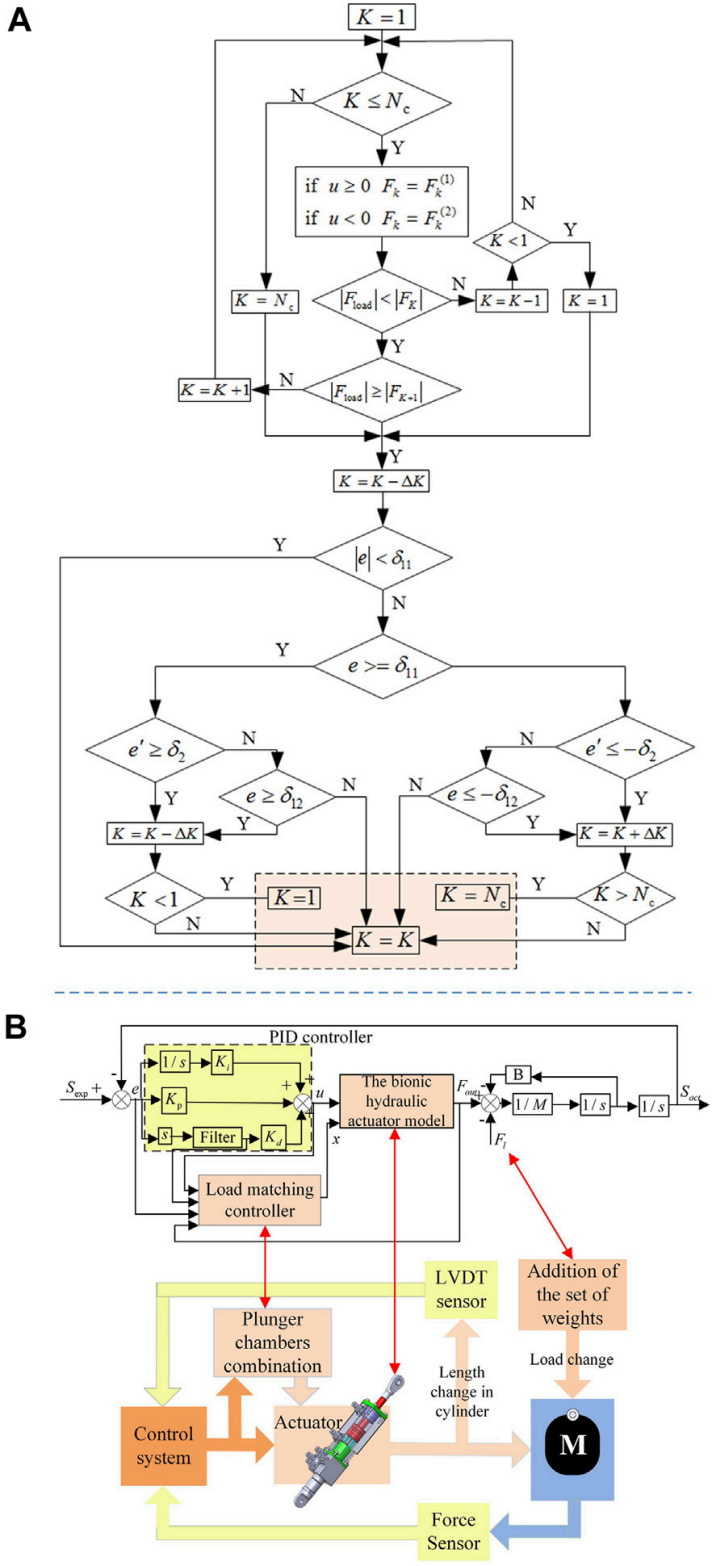
The BHA control system **(A)**. Load matching control process; **(B)**. The bionic hydraulic actuator control system.

Figure 5A demonstrates the load matching control process. K is the adjustment level shown in Table 1; ΔK is the adjustment level step; $N_{c}$ is the total number of adjustable levels, and for this design, $N_{c}=16$ ; u is the proportional servo valve opening (−1∼1); $F_{k}^{\left(1\right)}$ is the maximum output force of each level when $u=u_{\max}$ $F_{k}^{2}$ is the maximum output force of each level when $u=-u_{\max}$ ; e is the tracking error; and $e^{\prime }$ is the derivative of tracking error.

When the BHA starts to work, the maximum cross-sectional area is used as the effective action area, to ensure that the actuator has sufficient output force. The specific load matching control process is described as follows:

Step 1: According to the positive and negative of the control signal of the servo valve port, select the maximum output force $F_{k}$ of the corresponding level of multi-chamber variable section hydraulic c and compare it with the load force $F_{l}$ collected by the force sensor. If $\left|F_{l}\right|\geq \left|F_{k}\right|$ , the current effective action area selection is insufficient, and thus, the cross-sectional area level is reduced by one ( k=k−1 ). Then, return to the beginning. Nevertheless, If $\left|F_{l}\right|<\left|F_{k}\right|$ , then compare the size of $\left|F_{l}\right|$ and $\left|F_{k+1}\right|$ . If $\left|F_{l}\right|\geq \left|F_{k+1}\right|$ satisfied, the level K is the initially selected value, otherwise, add another level to K and return to the first step, and repeat the above process.

Step 2: When the level K is initially selected, compare the current tracking error e with the set value $\delta_{11}$ . $\left|e\right|<\delta_{11}$ indicates that the currently selected effective area is sufficient, and hence, the adjustment level K remains unchanged, and vice versa, the third step is carried out.

Step 3: Compare the tracking error derivative with the set value. When e is a positive value, $e^{\prime }\geq \delta_{2}$ reflects that the output force of the BHA is insufficient, and the error has a tendency to further expand, and therefore, the level K is reduced by ΔK levels. However, the minimum level K cannot go below 1; Oppositely, if $e^{\prime }<\delta_{2}$ , it indicates that the tracking error tends to shrink, it is necessary to compare the current error value e with the maximum allowable value $\delta_{12}$ . Here, if $e<\delta_{12}$ , keep the current adjustment level K unchanged, otherwise, reduce the level K by ΔK levels, to increase the output force of the actuator and the speed of error reduction. When e is a negative value, if $e^{\prime }\leq -\delta_{2}$ , it means that the selected effective area is too large, and the error has a tendency to expand, so the level K is increased by ΔK levels, but the minimum level K cannot exceed 16; If $e^{\prime }>-\delta_{2}$ , the tracking error has a tendency to shrink. However, it is again essential to compare the current error value e with the minimum allowable value $-\delta_{12}$ . If $e>-\delta_{12}$ , keep the current adjustment level K unchanged, otherwise, increase the level K by ΔK levels to accelerate the error reduction.

In this way, relying on the load matching control strategy of human muscles, a closed-loop control system for the multi-chamber BHAs is established, as highlighted in Figure 5B. Since the controller is not the focus of this work, the most widely used PID controller is used in this system. In Figure 5B, $s_{exp}$ is the expected displacement input; $s_{act}$ is the actual displacement output; M is the load mass; B is the damping coefficient; $K_{p}$ , $K_{i}$ and $K_{d}$ are the proportional, integral, and differential coefficients, respectively; x is the control signal of two-position three-way reversing valve; $F_{out}$ is the output load force of the BHA.

The control process of this system includes position servo closed-loop and load matching control loop. The position servo loop is the main control, and the specific control process of it is as follows: compare the expected displacement $s_{exp}$ with the actual displacement $s_{act}$ , and the obtained error e is used as the input of the PID controller. After the PID controller, the servo valve control signal u of the BHA is output to control the movement. When the BHA undergoes the position servo control, if the load changes, the load matching control loop starts to play its role, simultaneously. Based on the variation in load force, the best piston chamber combination mode is selected to reduce the throttling loss and improve energy efficiency. The specific load matching control process is as follows: input the BHA output force, servo valve control signal, and error and error derivative into the load matching controller. Following the load matching control calculation, the optimal control signal of the two-position three-way directional valve x is extracted, which then enters into the BHA model. Thus, different motor units are recruited to change the output force of the actuator, and hence, the control of the BHA is realized by the control system.

## 4 Efficiency Comparison of the Traditional and Proposed Actuator Based on a Human Arm

**FIGURE 6:**
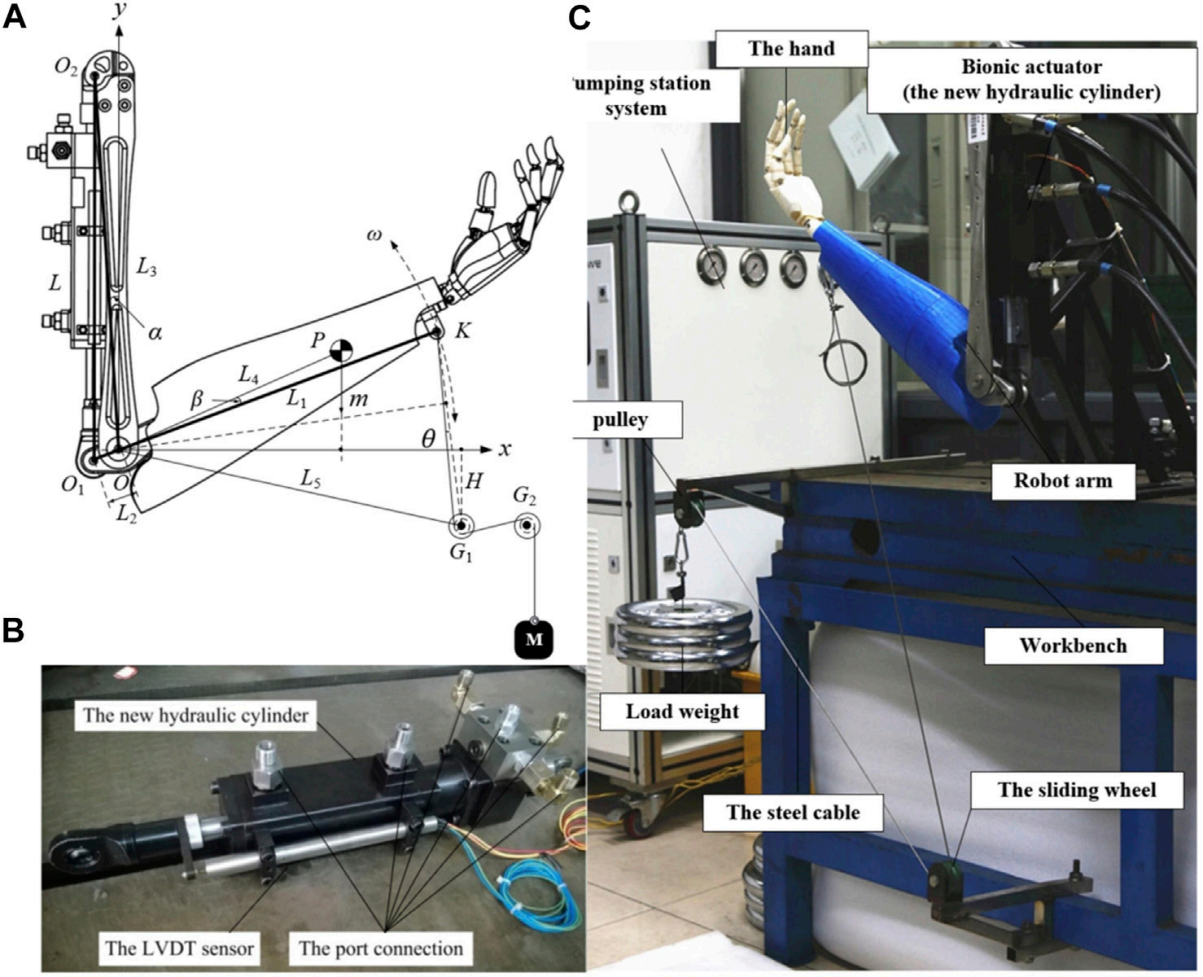
The experiments of the BHA; **(A)**. The geometry of the human arm; **(B)**. The real prototype of the proposed actuator; **(C)**. The experimental system using the proposed actuator.

To compare the efficiency of the hydraulic systems with the traditional actuator and the proposed actuator, a robotic platform of a human arm was designed, as shown in Figure 6A. The Cartesian coordinate system, Oxy, was located at Point O. Point P is the center of mass of the forearm and the hand. $G_{1}$ and $G_{2}$ are the two sliding wheels. α is the acute angle between the y-axis and the line $OO_{2}$ . β refers to the acute angle between OP and OK beelines. L denotes the cylinder length ( $O_{1}O_{2}$ ), and $L_{1}$ , $L_{2}$ , $L_{3}$ , $L_{4}$ and $L_{5}$ are the lengths of the beelines, OK , $OO_{1}$ , $OO_{2}$ , OP , and $OG_{1}$ , respectively. The term m is the mass of the forearm and the hand and M is the mass of the set of weights.

The torque of the BHA $M_{c}$ can be obtained as follows:

$$M_{c}=F_{l}\delta_{l}.$$ (14)

$$\delta_{l}=\frac{\sqrt{4L^{2}L_{2}^{2}-{\left(L^{2}+L_{2}^{2}-L_{3}^{2}\right)}^{2}}}{2L}.$$ (15)

Likewise, the torque of the load $M_{l}$ can be obtained as follows:

$$M_{l}=m\left(g\delta_{1}+\alpha_{1}L_{4}\right)+M\left(g+a_{2}\right)\delta_{2}.$$ (16)

$$\delta_{1}=L_{4}\cdot \sin\left(\arccos\left(\frac{L_{2}^{2}+L_{3}^{2}-L^{2}}{2L_{2}L_{3}}\right)+\alpha +\beta \right).$$ (17)

where $a_{1}$ and $a_{2}$ are the accelerated speeds of the centroid of the forearm and weight M , respectively. The torque arm $\delta_{2}$ is approximately equal to the distance from Point O to the straight line $KG_{1}$ because the angle θ was considerably small, and sin⁡θ≈0 .

$$\varphi =\pi -\alpha -\arccos\left(\frac{L_{2}^{2}+L_{3}^{2}-L^{2}}{2L_{2}L_{3}}\right).$$ (18)

Therefore, the torque arm $\delta_{2}$ is:

$$\delta_{2}=\frac{\left|HL_{1}\sin\varphi +\sqrt{L_{5}^{2}-H^{2}}L_{1}\cos\varphi \right|}{{\left(L_{1}\cos\varphi +H\right)}^{2}+{\left(\sqrt{L_{5}^{2}-H^{2}}-L_{1}\sin\varphi \right)}^{2}}.$$ (19)

Based on (17), the rotation speed of the forearm ω is:

$$\omega =\frac{d\varphi }{dt}=\frac{2L}{\sqrt{4L_{2}^{2}L_{3}^{2}-{\left(L_{2}^{2}+L_{3}^{2}-L^{2}\right)}^{2}}}\frac{dL}{dt}.$$ (20)

Here, the accelerated speeds $a_{1}$ $a_{1}$ can be obtained as:

$$a_{1}=L_{4}\cdot \frac{d\omega }{dt}.$$ (21)

$$a_{2}=L_{1}\cdot \frac{d\omega }{dt}\cdot \frac{\left|\sqrt{L_{5}^{2}-H^{2}}\cos\varphi +H\sin\varphi \right|}{\sqrt{{\left(L_{1}\sin\varphi -\sqrt{L_{5}^{2}-H^{2}}\right)}^{2}+{\left(L_{1}\cos\varphi +H\right)}^{2}}}.$$ (22)

Considering $M_{c}=M_{l}$ , the load of the cylinder $F_{l}$ can be obtained as:

$$F_{l}=f\left(L,\overset{\cdot }{L},\overset{\cdot \cdot }{L}\right).$$ (23)

Accordingly, the energy consumption of the load can be expressed as follows:

$$E_{l}=\int \overset{\cdot }{L}\cdot f\left(L,\overset{\cdot }{L},\overset{\cdot \cdot }{L}\right)dt.$$ (24)

Eventually, based on (4), 13, and 23, the efficiencies of the fluid power system of the forearm actuated by the typical actuator and the new actuator are expressed as:

$$\eta_{1}=\frac{E_{l}}{E_{1}}\times 100\%.$$ (25)

$$\eta_{2}=\frac{E_{l}}{E_{2}}\times 100\%.$$ (26)

## 5 Experiment

To validate that the hydraulic system with the new actuator was more efficient than that with the traditional actuator, a prototype of the new actuator was developed, as shown in Figure 6. Besides, the parameters of the manufactured prototype are listed in Table 2. As shown in Figure 6B, 6six oil ports and an LVDT sensor were used to measure the displacement of the cylinder.

**TABLE 2:** Paramenters of the experimental system.

Parameter	Value
Power source pressure (bar)	100
Tank pressure (bar)	5
Oil density (kg m-3)	875
Diameter of the main piston (mm)	32
Diameter of the main rod (mm)	16
Length of ${\text{L}}_{1}$ (mm)	353
Length of ${\text{L}}_{2}$ (mm)	28
Length of ${\text{L}}_{3}$ (mm)	378
Length of ${\text{L}}_{4}$ (mm)	275
Length of ${\text{L}}_{5}$ (mm)	664
Height of H (mm)	650
Angle α (rad)	0.064
Angle β (rad)	0.087
Mass of the forearm and hand m (kg)	1.5
Mass of the set of weights M (kg)	30

Additionally, an experimental system was built based on the robotic platform of a human arm, as shown in Figure 6C. The elbow joint was actuated using the new actuator, and one end of steel cable was affixed to the wrist. Moreover, the set of weights here is the applied load, which was attached to the other end of the steel cable. When the elbow joint rotated, the set of weights moved up and down. The detailed parameters of the experimental system are listed in Table 2.

**FIGURE 7:**
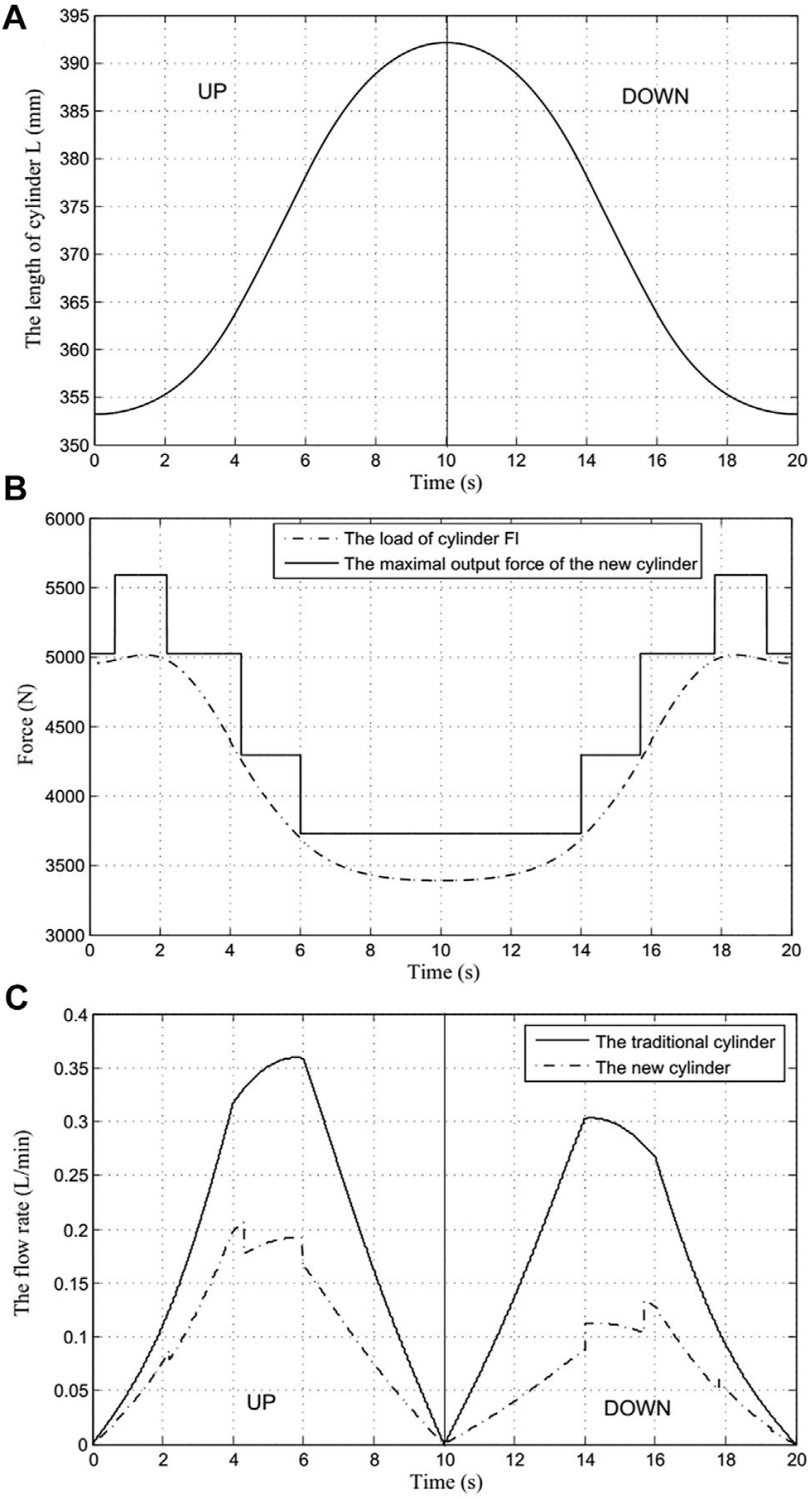
The results of the experiment **(A)**. Displacement of the cylinder; **(B)**. Load and matching output force of the proposed actuator; **(C)**. Flow rate supplied by the hydraulic system.

A periodic working process of the robotic arm was defined, consisting of two processes: rod extension and retraction. Figure 7A shows the displacement of the actuator during one periodic time. According to the analysis provided in Section 4, the load of the actuator was determined by the torque arm, indicated by the dash-dotted line in Figure 7B. Figure 7B displays the load and the matching output force of the proposed actuator. The solid line in Figure 7B represents the maximal output force of the new actuator, while the dash-dotted line represents the load of the actuator. As evident from Figure 7B, the maximal output force of the new actuator can be matched to the load by changing the effective area, according to the principle of the actuator described in Section 3. When the input load force increases, the effective area of the BHA is increased through the recruitment of the motion unit, and consequently, the maximum output force of the BHA is also increased. Contrarily, when the input load force is reduced, the effective cross-sectional area is also reduced due to the change of the recruited motion unit combination, thereby reducing the maximum output force of the BHA. On the other hand, the experiment conducted with the traditional hydraulic cylinder indicates that the maximal output force cannot be adjusted because the effective area is constant. These results demonstrate that the BHA proposed herein has good controllability under different load forces.

Notably, the forearm can be driven down by the gravity of the set of weights; that is, the pump source only needs to supply the low-pressure oil. Therefore, the energy is consumed only when the set of weights is actuated upward. The energy consumption of the load can be calculated based on (24), and El = 157.2 J.

Furthermore, Figure 7C shows the flow rate supplied by the hydraulic systems comprising the traditional and proposed cylinders. Evidently, the flow rate supplied by the traditional cylinder hydraulic system was significantly high, as shown in Figure 7C. Using (4) and 13, the energy supplied by the hydraulic systems with the traditional cylinder and proposed cylinder can be calculated as $E_{1}=313.1J$ and $E_{2}=170J$ , respectively. Correspondingly, utilizing (25) and (26), the efficiency of the hydraulic systems with the traditional and proposed actuators was calculated as $\eta_{1}=50.2\text{\%}$ and $\eta_{2}=92.5\text{\%}$ , respectively. These efficiency results clearly demonstrate that the new (BHA) system can indeed improve the overall energy efficiency of the robot.

**FIGURE 8:**
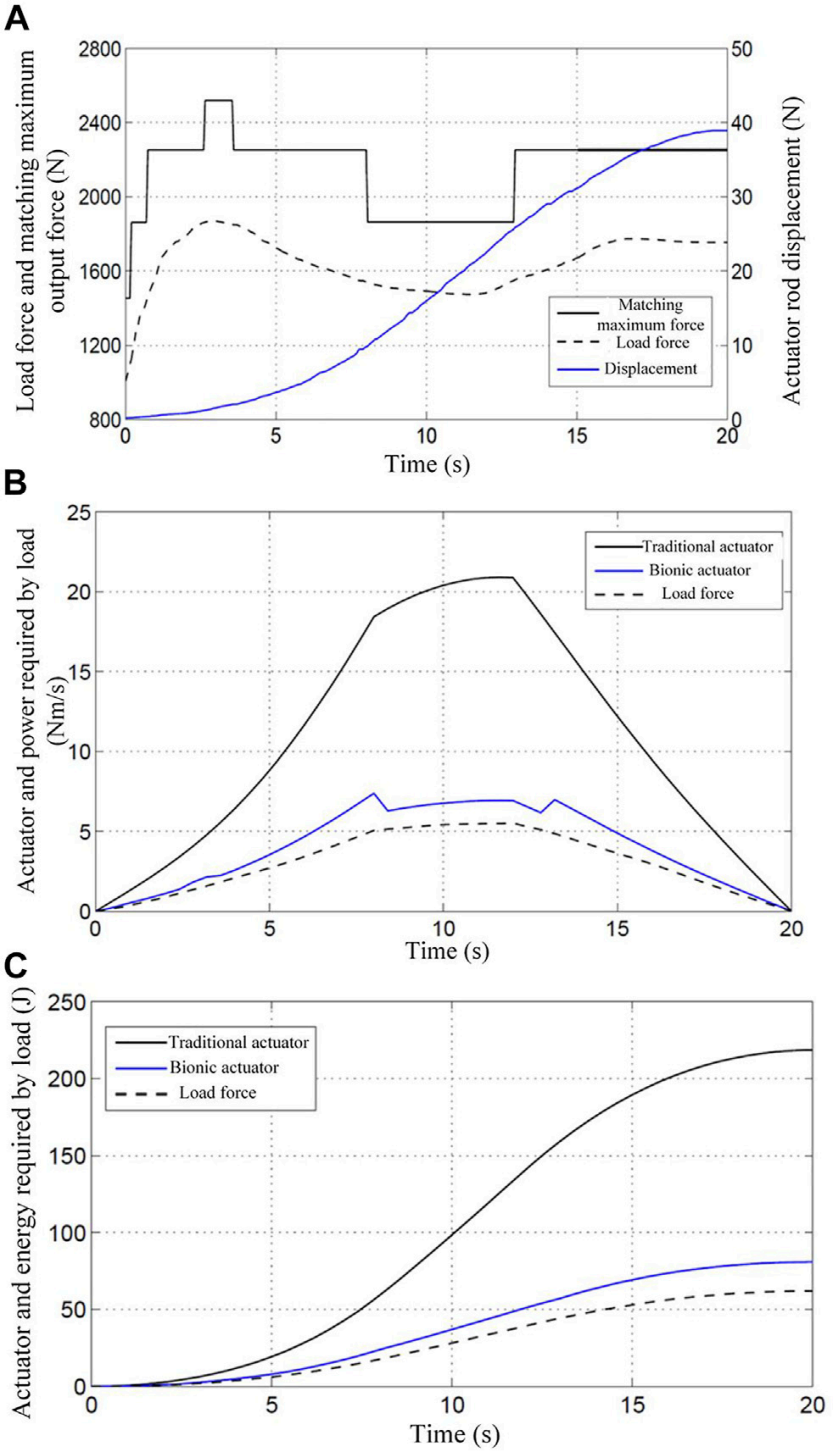
The results of the experiment under a load of 10 kg **(A)** 10Kg load force matching; **(B)** 10Kg load power consumption curve; **(C)** 10Kg load energy consumption curve.

To further analyze and verify the efficiency of the multi-chamber BHA, the load matching and energy utilization of the actuator during the lifting of heavy objects by the robotic arm are analyzed experimentally. By comparing the energy utilization rate of the proposed BHA with the CHA, it is verified that the new BHA can improve the energy utilization efficiency. The experiment simulates the process of lifting a heavy object in humans. According to the rotation angle of the elbow joint, the robotic arm starts to accelerate uniformly, then decelerates uniformly, and finally moves at a uniform speed, during the process of lifting the heavy object. During this process, the pressure provided by the pump source is 7 Mpa. Figure 8A shows the load force matching during the lifting of 10 KG weight by the robot arm. It is obvious from the figure that the BHA can adjust the number and size of the recruited motor units in real-time according to the load change, which in turn changes the amount of its output force.

Based on the principle of designed BHA, if all the motion units of the actuator are recruited, the BHA is simply equivalent to the CHA. In order to facilitate the experiments and comparisons, this approach is used to simulate the CHA for experimentation. From Figure 8B, the power consumed by the CHA is much higher than the load power, while the power consumed by the BHA is only slightly higher than the load power. The energy consumed by the extraction of heavy objects (10 Kg) can be obtained through relevant calculations of the robot arm. As shown in Figure 8C, the total energy required for one extraction is 61.9J, whereas the total energy consumed by the BHA is 80.9J, while that consumed by the CHA is 218.6J. Using the formula, the energy utilization rate of the BHA and the CHA can be obtained as $\eta_{1}=76.6\text{\%}$ and $\eta_{2}=28.3\text{\%}$ , respectively.

**FIGURE 9:**
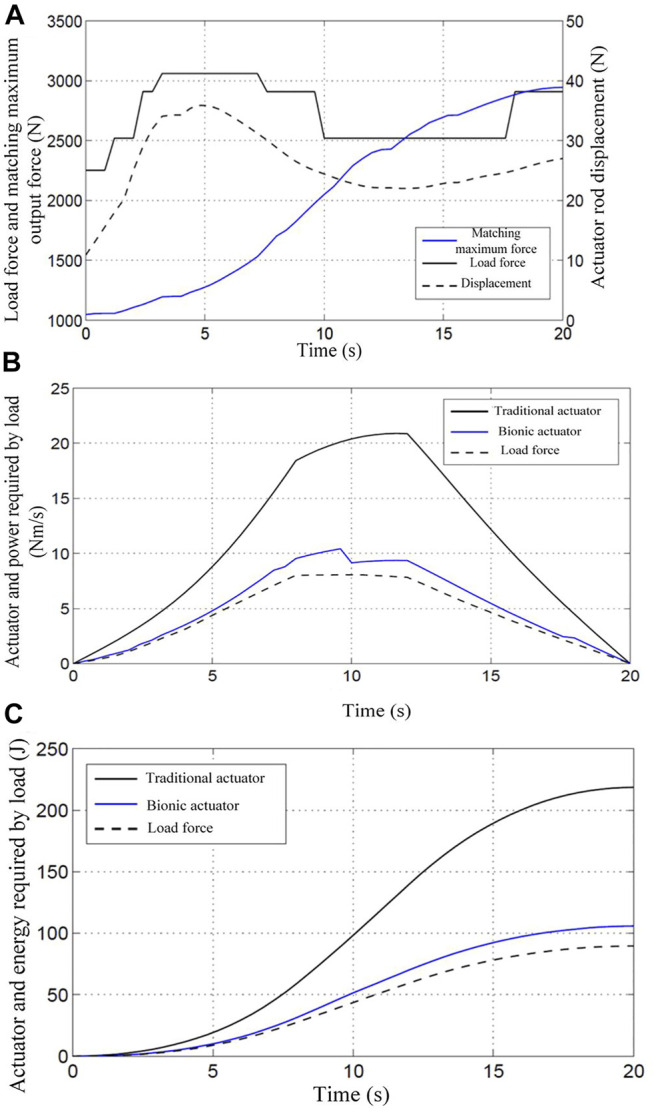
The results of the experiment under a load of 15 kg **(A)** 15Kg load force matching; **(B)** 15Kg load power consumption curve; **(C)** 15Kg load energy consumption curve.

In addition, the weight is increased from 10 Kg to 15 Kg, and the above experiment is repeated. Figure 9A, Figure 9B, and Figure 9C illustrate the load force matching, power consumption curve, and load energy consumption curve, respectively, in the process of lifting the 15 Kg weight by the robot arm.

Similarly, the load power, the power consumed by BHA, and the power consumed by CHA can be obtained using the relevant formula, as shown in Figure 9B. Besides, it is evident from the figure that the power consumed by the CHA is much higher than the load power, while the power consumed by the BHA is only slightly higher than the load power. Moreover, the energy consumed by the robotic arm to extract 15 Kg is calculated using the formula and presented in Figure 9C. The total energy required for the extraction is 89.6J, and the total energy consumption of the BHA is 105.8J, while that of the CHA is still 218.6J. Similar to the case of 10 kg load, the energy utilization rate of the BHA can be obtained using the formula as $\eta_{1}=84\text{.}7\text{\%}$ , whereas the energy utilization rate of the CHAs is $\eta_{2}=40\text{.}6\text{\%}$ .

It can be seen from the above experiments that under the conditions of two different loads (10 Kg and 15 Kg), the CHA consumed the same amount of energy. This is because the energy consumed by the CHA is only related to the system oil supply pressure and input flow. Therefore, for traditional actuators, the smaller the load, the lower the energy utilization. The BHA, on the other hand, can adjust the flow rate of the system input by adjusting the number and size of the motion units involved in the work, thereby improving the energy utilization. At the same time, experiments show that, with the change in load force, the new BHA changes the output force by recruiting different motion units, which indicates good controllability.

## 6 Conclusion and Future Work

In this study, a new type of BHA was designed by imitating the driving principle and configuration of human muscles, in view of their excellent structural and energy-saving properties, to improve the efficiency of the hydraulic system of bionic robots. This work focused on the comprehensive design and application of the new actuator with a controllable effective area. The impact of the traditional and the new actuator on the efficiency of a hydraulic system based on a robotic hand was compared. Initially, the principle of the new actuator was presented, following which the prototype was developed. A robotic arm was designed and used to demonstrate that the proposed actuator could significantly improve the efficiency of a hydraulic system with a variable load. The experimental results reveal that the proposed actuator can substantially improve the efficiency of the hydraulic system for mobile multi-joint legged robots. Simultaneously, the newly designed BHA can be deployed for mobile bionic robots with time-variant load force.

In the proposed multi-chamber BHA structure, since the chambers are of different sizes and are not symmetrically distributed, the total force of the actuator hydraulic cylinder is not at the center of the main piston rod. Consequently, the combined force of several piston rods can cause the main piston rod to deflect. Accordingly, an optimization method can be considered to mitigate this problem (Xue et al., 2015).

In future research, we will integrate the valve body and BHA structure to further reduce the overall volume and mass of the BHA structure. Simultaneously, we will explore other structural forms, control algorithms or completely different driving methods.

## Data Availability

The original contributions presented in the study are included in the article/Supplementary Material, further inquiries can be directed to the corresponding authors.
